# Wakeful rest compared to vigilance reduces intrusive but not deliberate memory for traumatic videos

**DOI:** 10.1038/s41598-019-49634-8

**Published:** 2019-09-16

**Authors:** Lone D. Hørlyck, James A. Bisby, John A. King, Neil Burgess

**Affiliations:** 1 UCL Institute of Cognitive Neuroscience, University College London, London, UK; 2 UCL Institute of Neurology, University College London, London, UK; 3 Department of Clinical and Health Psychology, University College London, London, UK

**Keywords:** Consolidation, Long-term memory, Human behaviour

## Abstract

Intrusive memories are prominent features of post-traumatic stress disorder, but the mechanisms supporting their development, and their relationship to deliberate memories, are subject to competing theories. Are they strengthened examples of a unitary memory system, or fragmented representations lacking aspects of healthy memories? Given the importance of post-encoding processing in memory consolidation, we investigated the effects of a brief wakeful rest compared to a vigilance task immediately after the encoding of traumatic material on subsequent intrusive and deliberate memory. Across two experiments, participants watched emotionally negative film clips immediately followed by a brief wakeful rest or a simple vigilance (0-back) task. Brief wakeful rest had distinct effects on memory compared to the 0-back task, reducing intrusive memory frequency but not changing deliberate memory performance. These differential effects suggest that intrusive memory and deliberate memory reflect dissociable systems, arguing against unitary accounts. Our findings highlight the importance of post-encoding processing in the consolidation of traumatic material and the development of intrusive memories and provide a new perspective for interpreting mechanisms of therapeutic intervention.

## Introduction

Following exposure to severe trauma, people may experience memory disturbances in the form of distressing images that involuntarily enter consciousness, as seen in post-traumatic stress disorder (PTSD)^1^. Most models of intrusive memories identify the period immediately after the trauma as crucial in determining the subsequent re-experience of traumatic imagery^2–5^. Thus, understanding the factors that interact with post-trauma processing is key both to understanding the mechanisms that support the development and maintenance of memory intrusions and to informing potential psychological treatment strategies^6^.

Newly formed memories are thought to undergo consolidation as they are strengthened and stabilised^7,8^. Memories are particularly sensitive during this transient window and are susceptible to interference from a broad range of influences, including amnestic agents and retroactively interfering stimuli^9^. For instance, when naturalistic movie clips are immediately followed by new interfering stimuli, memory for the initial clips is reduced, corresponding to an attenuation of post-encoding activity in memory-related brain regions^10,11^. However, it remains unclear how the events that unfold in the immediate aftermath of a trauma affect subsequent memory and related symptomatology.

Studies using an analogue trauma paradigm, during which traumatic material is viewed and subsequent intrusions collected for one week, show that unrelated tasks performed after encoding can alter intrusive memory reports^12,13^. Interestingly, the type of task performed after encoding is important, with visuo-spatial tasks such as playing Tetris or complex finger-tapping carried out during or after encoding found to reduce the number of intrusions^14–16^ whereas others, such as carrying out verbal tasks can increase their occurrence^17,18^. In contrast, deliberate memory for the traumatic material is often unaffected by such post-encoding manipulations^15,16^. Whilst a consolidation window offers a period in which trauma memories might be altered to ameliorate or accentuate symptoms, the effects of different tasks or natural behaviours on intrusive imagery and deliberate memory are not well understood.

A unitary view of memory for trauma^4,5^ corresponds to the idea of a single medial temporal lobe declarative memory system^19^ and proposes that the presence of highly salient emotional content leads to stronger long-term declarative memories^20^. This account proposes that both intrusive and deliberate memory following a traumatic experience reflect operation of the same system.

By contrast, a dual representation view^2,21,22^ corresponds to the idea of multiple memory systems, in which associative contextual representations are supported by the hippocampus while sensory and affective representations rely on the amygdala and sensory/interoceptive neocortex^23,24^. In this account, deliberate recollection of the event is mediated by associative and contextual representations in the hippocampal system, which supports retrieval of sensory and affective content in a coherent and controlled manner. However, the presence of highly salient emotional content up-modulates the amygdala system and down-modulates the hippocampal system, allowing formation of strong sensory and affective representations with impoverished associative contextual representations. Thus, sensory affective representations can be reactivated involuntarily by environmental cues and re-experienced outside of their associated context as intrusive imagery^3^.

Hence, within this view, there are potentially two routes to reducing the occurrence of memory intrusions: either to disrupt perceptual/emotional processing of the trauma or, alternatively, to enhance contextual processing of the trauma. Consistent with the former idea, previous studies have shown that disrupting post-encoding consolidation of perceptual aspects of the trauma (e.g. by playing the computer game Tetris) is associated with a reduction in intrusions^15,25^. Here, we investigate the latter alternative proposed by the dual representation theory, namely that enhancing contextual consolidation will also reduce memory intrusions.

Under a unitary view, disruption or facilitation of memory consolidation would be expected to have similar effects on both deliberate and intrusive memory reports. In contrast, a dual representation view predicts dissociable effects of manipulating consolidation depending on the specific memory representation targeted. Thus, disrupting the consolidation of sensory representations, e.g. by playing Tetris^14,15^, should reduce intrusive memory frequency under both views. However, selective disruption of hippocampal consolidation should reduce intrusions under a unitary view but increase them under a dual representation account. Further, a unitary account would predict that facilitation of hippocampal consolidation should enhance both intrusive and deliberate memory, whilst a dual representation view predicts fewer intrusive memory occurrences.

Post-encoding manipulations aimed at reducing external sensory information can improve subsequent declarative memory. For example, a brief wakeful rest period following new learning of neutral information improves memory retention both in the short and long-term compared to a simple ‘spot the difference’ task^26,27^. Rest has also been found to facilitate consolidation and subsequent memory performance for stressful stimuli^28,29^. Studies have suggested that this relative increase in memory following rest is not dependent on active rehearsal and appears to rely on offline processes^26^. Such findings are consistent with studies investigating memory consolidation during sleep, where reinstatement of activity present during encoding during sleep is associated with better subsequent memory^30^. These processes may be related to hippocampal ‘replay’ observed in rodents that is believed to facilitate memory consolidation^31,32^. Thus, brief wakeful rest periods following encoding are thought to facilitate hippocampal consolidation processes, with post-encoding increases in hippocampal activity^10,11,33^ and functional connectivity between the hippocampus and cortical structures^28,29,34^ found to correlate with subsequent memory performance. However, it is unclear how facilitation of consolidation by a brief wakeful rest would impact subsequent intrusive imagery for traumatic material and what the relationship between intrusive and deliberate memory would be.

Here, we investigated how providing a brief wakeful rest in the immediate aftermath of viewing traumatic material would affect subsequent memory compared to a vigilance (0-back) task. The 0-back task requires sustained attention but has no working memory load and has been shown to not affect the amount of amygdala or hippocampal activity response following the viewing of stressful videos^35^. In two studies, we examined whether deliberate memory for the negative events would be improved, and whether intrusive imagery would be increased (as predicted by unitary accounts^4,5^) or reduced (as predicted by dual representation accounts^2,21,22^) by the wakeful rest compared to the 0-back task. For this purpose, participants watched a series of short audio-visual video clips comprising only negative (Experiment 1) or negative and neutral (Experiment 2) events and were allocated to either a brief 10 minute period of wakeful rest or completing a 0-back task following encoding. Over the week following encoding, participants kept a diary of memory intrusions related to the videos and returned for a memory test on Day 8.

## Results

### Experiment 1

#### Self-report questionnaires

See Table 1 for a breakdown of ratings from questionnaires. Analysis of trait anxiety scores showed no baseline differences between the brief wakeful rest and vigilance task groups (t(38) = 0.44, p = 0.662, d = 0.14, equal variances not assumed). Analysis of state anxiety using a 2 × 2 mixed ANOVA (group × time) showed a significant main effect of time (F(1,38) = 124.00, p < 0.001, η² = 0.77) reflecting increased state anxiety from pre- to post-film. There was no significant main effect of group (F(1,38) = 2.04, p = 0.161, η² < 0.01) or group × time interaction (F(1,38) = 0.12, p = 0.736, η² < 0.01).

**Table 1:** Means (SD) of self-report ratings in Experiment 1.

	Brief Wakeful Rest *n* = 21		Vigilance Task *n* = 19	
	Pre	Post	Pre	Post
Trait anxiety	41.68 (11.98)	n/a	39.58 (8.19)	n/a
State anxiety	30.35 (6.75)	52.20 (12.91)	33.21 (6.05)	55.68 (11.93)
Positive affect	29.70 (7.54)	25.60 (6.66)	29.95 (8.28)	22.32 (5.77)
Negative affect	11.75 (1.77)	22.85 (7.35)	13.21 (2.51)	23.84 (7.41)
DSSQ_Total_	n/a	50.60 (16.04)	n/a	53.53 (15.60)
DSSQ_Trauma_	n/a	18.20 (7.27)	n/a	18.11 (8.03)

Analysis of positive affect showed a main effect of time (F(1,38) = 44.83, p < 0.001, η² = 0.54) with a decrease in positive affect from pre- to post-film. There was no significant main effect of group (F(1,38) = 0.31, p = 0.590, η² = 0.01) or group × time interaction (F(1,38) = 3.80, p = 0.0590, η² = 0.09). Similarly, analysis of negative affect also showed a main effect of time (F(1,38) = 104.67, p < 0.001, η² = 0.73) due to an increase in negative affect from pre- to post-film and no main effect of group (F(1,38) = 1.10, p = 0.302, η² = 0.28) or group × time interaction (F(1,38) < 0.01, p = 0.995, η² < 0.01).

Finally, global DSSQ scores indicated that there were no differences between the two groups in engagement, stress and worry during wakeful rest and completion of the vigilance task, t(37) = 0.58, p = 0.567, d = 0.18. Likewise, there were no differences between groups in the degree to which participants had thoughts related to the trauma videos during rest or vigilance task, as reflected in the DSSQ trauma-related thoughts sub-score, t(37) = 0.04, p = 0.969, d = 0.01.

#### Comparing memory types

To directly assess differences between intrusive and deliberate memory, we performed a 2 × 2 ANOVA with condition as a between group factor (wakeful rest, vigilance task) and memory type (intrusive memory, deliberate memory) as a within participant factor. This analysis showed a significant condition × memory type interaction, F(1,36) = 5.10, p = 0.030, η^2^ = 0.12, indicating that the conditions (wakeful rest and vigilance task) had different effects on intrusive and deliberate memory.

#### Memory intrusions

**Figure 1:**
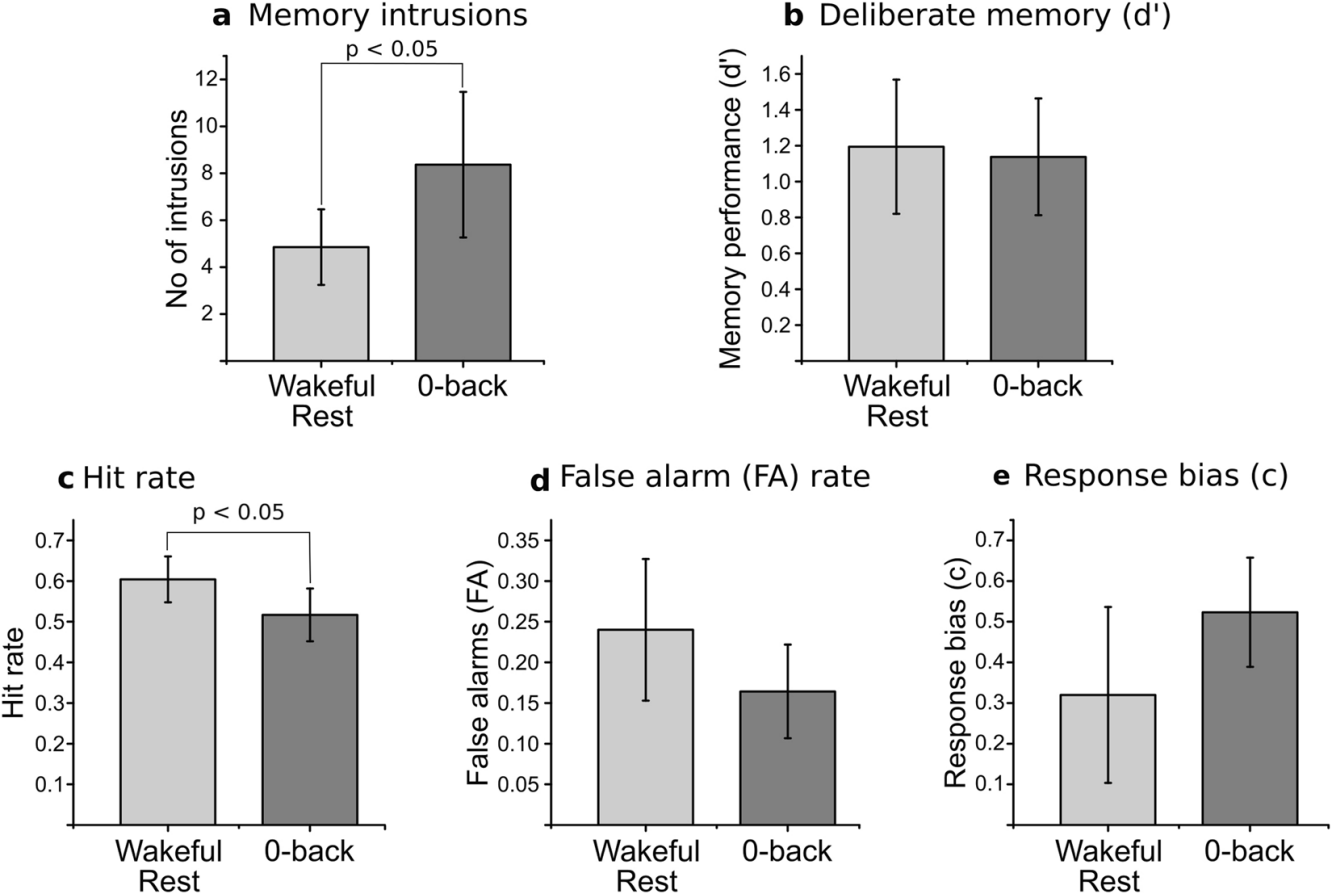
Experiment 1 results. Above: Number of intrusions reported over the 1 week after viewing the trauma film (**a**) and performance in recognising images during the deliberate memory task at follow-up (**b**) in brief wakeful rest or 0-back vigilance task groups. Below: Hit rate (**c**); false alarm rate (**d**) and response bias (**e**) in the recognition memory test for the wakeful rest and 0-back vigilance task groups. Bars show means with 95% confidence intervals (CI).

Prior to analysis, intrusive memory data were log-transformed due to evidence of non-normality (Shapiro-Wilk, p’s < 0.001). Subsequent analysis showed a significant difference between conditions (t(37) = 2.04, p = 0.049, d = 0.65) with fewer intrusive memories reported by participants given brief wakeful rest (M = 4.85, SD = 3.45) following the trauma film compared to participants given a vigilance task (M = 8.34, SD = 6.44) (Fig. 1a).

#### Deliberate memory

Performance on the deliberate memory test carried out at follow up on day-8 was assessed by analysing d’ scores, taking both hits and false alarms into account (Fig. 1b). An independent samples t-test showed no significant difference between wakeful rest (M = 1.19, SD = 0.78) and vigilance task (M = 1.14, SD = 0.68) groups on d’ scores, t(36) = 0.24, p = 0.813, d = 0.08. There was a significant increase in the proportion of hits following wakeful rest (M = 0.60, SD = 0.12) compared to the vigilance task (M = 0.52, SD = 0.13), t(36) = 2.13, p < 0.040, d = 0.69 (Fig. 1c). By visual inspection, there was also a higher false alarm rate in the wakeful rest group (M = 0.24, SD = 0.18) compared to the vigilance task (M = 0.16, SD = 0.12), but this difference did not reach significance, t(36) = 1.53, p = 0.136, d = 0.50 (Fig. 1d). There was no difference between the two groups on a measure of response bias (c), t(36) = 1.64, p = 0.109, d = 0.53 (Fig. 1e).

### Experiment 2

Results from Experiment 1 showed that a brief wakeful rest versus a 0-back task in the immediate aftermath of a trauma film had distinct effects on intrusive and deliberate memory. A brief wakeful rest after watching aversive clips reduced intrusive memories over the following week compared to the 0-back task, while there were no differences in deliberate memory after one week between the two groups. The lack of a difference between groups in deliberate memory appears contradictory to previous findings showing increased deliberate memory performance following rest compared to simple cognitive tasks^26^. However, the significant increase in hit rate with wakeful rest compared to the vigilance task might have driven an increase in d’ had it not been for the numerical increase in false alarms in the wakeful rest group.

We next attempted to replicate and extend these findings by adopting a repeated measures design in which participants performed both conditions (brief wakeful rest and vigilance task) on separate occasions. Further, as we did not see a facilitation effect of wakeful rest compared to the 0-back task on deliberate memory performance, we included a mixture of neutral and negative clips at encoding in an attempt to tease apart the effects of rest on consolidation processes for neutral and negative events. We also utilised an intrusion provocation task^36^ as a second measure of intrusive memory collected on day 8, in which participants were cued with blurred static images to trigger related intrusive memories.

#### Self-report questionnaires

See Table 2 for a breakdown of ratings from questionnaires. For state anxiety (STAI), a 2 × 2 repeated measures ANOVA with condition (brief wakeful rest, vigilance task) and time (pre-, post-encoding) as within participant factors showed a significant main effect of time (F(1,28) = 131.42, p < 0.001, η² < 0.82) with an increase in anxiety from before to after the trauma films. There was no main effect of condition (F(1,28) = 0.25, p = 0.620, η² < 0.01) or condition × time interaction (F(1,28) = 2.39, p = 0.134, η² = 0.08).

**Table 2:** Means (SD) of self-report ratings in Experiment 2.

	Brief Wakeful Rest		Vigilance Task	
	Pre	Post	Pre	Post
Trait anxiety	41.23 (11.35)	n/a	n/a	n/a
State anxiety	31.58 (8.31)	47.97 (11.31)	35.08 (9.69)	49.79 (10.19)
Positive affect	30.36 (7.49)	26.81 (7.62)	28.29 (6.64)	23.97 (6.42)
Negative affect	13.83 (4.07)	20.28 (8.28)	13.65 (4.17)	21.44 (8.37)
DSSQ_Total_	n/a	47.71 (12.32)	n/a	46.12 (16.23)
DSSQ_Trauma_	n/a	16.81 (7.25)	n/a	14.15 (7.87)

Analysis of positive affect using a similar 2 × 2 ANOVA showed a main effect of time (F(1,28) = 18.73, p < 0.001, η² = 0.40), reflecting decreases in positive affect from before to after viewing the trauma films. We also saw a main effect of condition (F(1,28) = 7.59, p = 0.010, η² = 0.21) with slightly greater positive affect scores in the brief wakeful rest compared to vigilance task condition. Importantly, the condition × time interaction was not significant (F(1,28) = 0.25, p = 0.619, η² < 0.01), showing that groups did not differ in the amount of change in positive affect initiated by the trauma films. For negative affect, analysis using a similar 2 × 2 ANOVA showed a main effect of time (F(1,28) = 46.57, p < 0.001, η² = 0.63) due to increased negative affect from before to after viewing the trauma films. The main effect of condition (F(1,28) = 0.08, p = 0.779, η² < 0.01) and condition × time interaction (F(1,28) = 0.68, p = 0.418, η² = 0.02) were not significant.

For the DSSQ total score reflecting engagement, distress and worry during rest or the vigilance task, there was no difference between the wakeful rest and vigilance task conditions, t(31) = 0.61, p = 0.548, d = 0.11 For the trauma-related DSSQ score however, participants had higher scores in the wakeful rest condition (M = 16.81, SD = 7.25) than in the vigilance task condition (M = 14.15, SD = 7.87), t(31) = 2.30, p = 0.028, d = 0.41.

#### Comparing memory types

Similar to Experiment 1, we performed a 2 × 2 repeated measures ANOVA on memory for negative material, with memory type (diary intrusions, recognition memory) and condition (wakeful rest, vigilance task) as within-participant factors. Analysis revealed a significant type × condition interaction, F(1,35) = 5.63, p = 0.023, η^2^ = 0.138, indicating that the wakeful rest and vigilance task interventions had different effects on intrusive memories and deliberate recognition memory.

#### Memory intrusions

**Figure 2:**
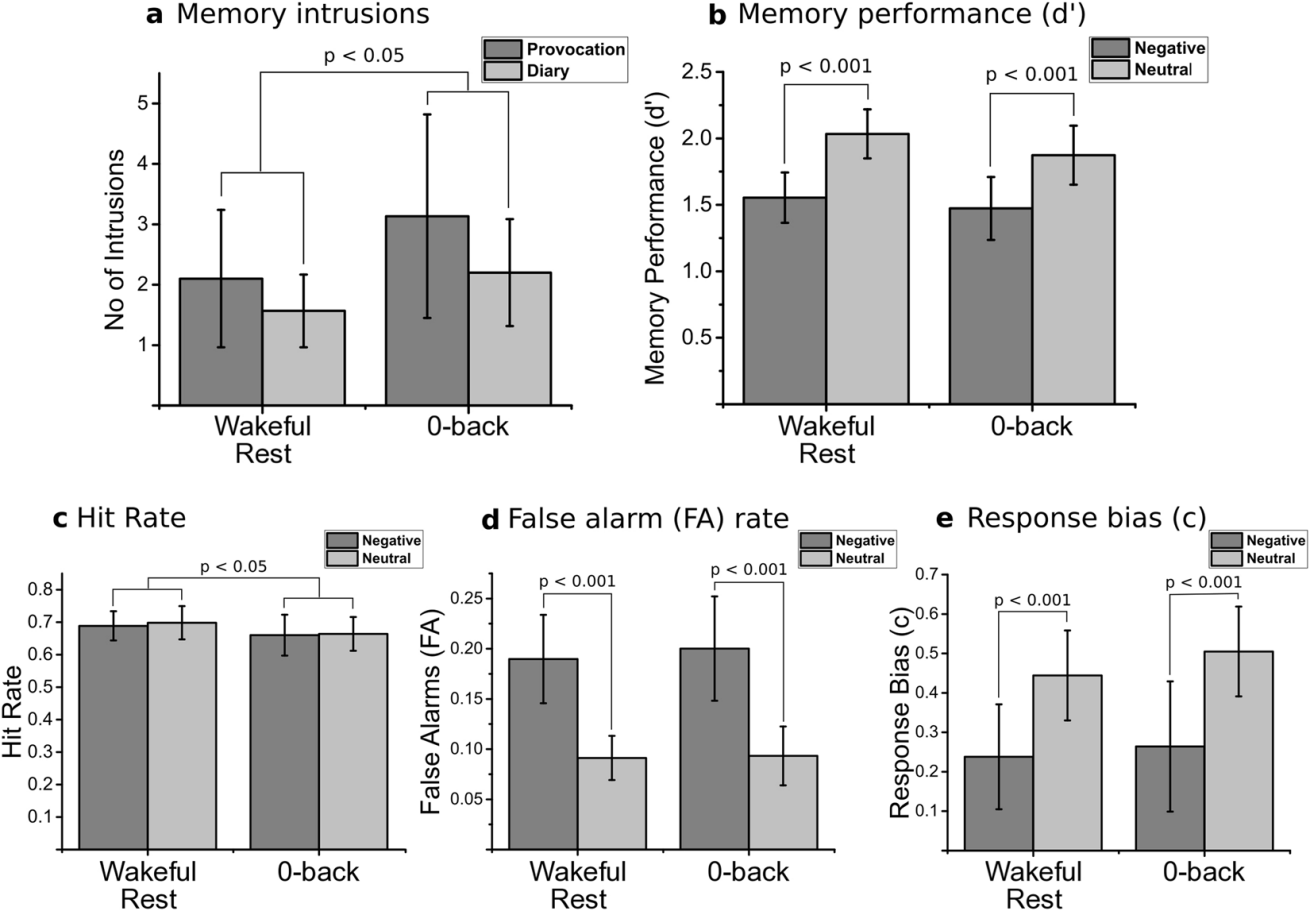
Experiment 2 results. Above: Number of reported intrusions in the 7-day diary and the intrusion provocation task (**a**) and recognition memory performance for neutral and negative items for the brief wakeful rest and 0-back vigilance task conditions (**b**). Below: Hit rate (**c**), false alarm rate (**d**) and response bias (**e**) in the recognition memory test for neutral and negative items for each of the two groups (wakeful rest and 0-back vigilance task). Bars represent means and 95% CIs.

Intrusion data were transformed prior to analyses using a log-transform as assumptions of normality were violated (Shapiro-Wilk, p’s < 0.05). As memory intrusions were recorded using a diary over 1 week and also at follow up using an intrusion provocation task (where participants were shown blurry pictures from the videos and subsequently asked to write down if they had any memory intrusions in the minutes following picture presentation), we analysed log-transformed intrusion data using a 2 × 2 repeated measures ANOVA with condition (brief wakeful rest, vigilance task) and test (diary, provocation) entered as within participant factors. Replicating the results from Experiment 1, this analysis showed a significant main effect of condition (F(1,29) = 6.03, p = 0.020, η^2^ = 0.17) with fewer intrusions in the brief wakeful rest condition compared to the vigilance condition (Fig. 2a). This effect was driven by relative reductions in both diary intrusions (wakeful rest: M = 1.97, SD = 2.76; 0-back: M = 2.76, SD = 3.96) and intrusion provocation task intrusions (wakeful rest: M = 1.44, SD = 1.56; 0-back: M = 2.18, SD = 2.28). There was no main effect of test (F(1,29) = 0.15, p = 0.703, η^2^ < 0.01) or condition × test interaction (F(1,29) = 0.11, p = 0.742, η^2^ < 0.01).

#### Deliberate memory

Similar to Experiment 1, recognition memory performance was analysed by calculating d’ scores. Deliberate memory performance was then assessed using a 2 × 2 repeated measures ANOVA on d’ values with video valence (neutral, negative) and condition (wakeful rest, vigilance task) as within-participant factors. Analysis showed a significant main effect of valence, F(1,39) = 44.95, p < 0.001, η^2^ = 0.54, due to higher d’ values for neutral compared to negative clips (Fig. 2b). The main effect of condition, F(1,39) = 1.73, p = 0.196, η^2^ = 0.03 was not significant, reflecting similar scores between conditions for both neutral (wakeful rest: M = 2.03, SD = 0.57; 0-back: M = 1.87, SD = 0.69) and negative (wakeful rest: M = 2.03, SD = 0.57; 0-back: M = 1.56, SD = 0.59) scores. Finally, the valence × condition interaction, F(1,39) = 0.47, p = 0.495, η^2^ = 0.01 was not significant.

For analysis of the proportion of hits, a 2 × 2 repeated measures ANOVA with valence (neutral, negative) and condition (wakeful rest, vigilance task) was carried out and revealed a main effect of condition, F(1,39) = 4.20, p = 0.047, η^2^ = 0.10. This effect was due to a higher hit rate in the wakeful rest condition compared to the vigilance task condition in both the neutral (wakeful rest: M = 0.70, SD = 0.16; 0-back: M = 0.66, SD = 0.16) and negative videos (wakeful rest: M = 0.69, SD = 0.14; 0-back: M = 0.66, SD = 0.20; Fig. 2c). The main effect of valence, F(1,39) = 0.12, p = 729, η^2^ < 0.01 and the valence × condition interaction F(1,39) = 0.05, p = 0.833, η^2^ < 0.01, were both non-significant.

An equivalent 2 × 2 repeated measures ANOVA performed on the false alarm rates revealed a main effect of valence, F(1,39) = 44.36, p < 0.001, η^2^ = 0.53, reflecting that across both conditions, participants had more false alarms for negative items compared to neutral items (Fig. 2d). The main effect of condition, F(1,39) = 0.13, p = 0.718, η^2^ < 0.01, and the valence × condition interaction F(1,39) = 0.13, p = 0.716, η^2^ < 0.01 were non-significant. Finally, a 2 × 2 repeated measures ANOVA conducted with response bias scores (c-values) showed a main effect of valence, F(1,39) = 20.28, p < 0.001, η^2^ = 0.34, which was due to higher response bias in the neutral valence compared to the negative valence, across conditions (Fig. 2e). The main effect of condition F(1,39) = 0.63, p = 0.432, η^2^ = 0.02, and the valence × condition interaction F(1,39) = 0.22, p = 0.643, η^2^ < 0.01 were non-significant.

## Discussion

The present study examined whether giving participants a brief wakeful rest in the immediate aftermath of viewing traumatic material would alter deliberate and intrusive memory for the experience compared to when participants carried out a 0-back task. We show that brief wakeful rest was associated with fewer intrusive memory reports over the week following the trauma film, compared to a simple vigilance task administered for the same duration. In contrast, deliberate memory for the footage tested after 1 week did not differ between the two conditions. Our results suggest that compared to a vigilance task, brief wakeful rest affects memory systems in distinct ways, reducing intrusive re-experiencing compared to the vigilance task, whilst sparing deliberate memory recall. These selective effects suggest that dissociable memory systems contribute to deliberate and intrusive memory and can be targeted independently.

Across experiments, brief wakeful rest was associated with fewer intrusive memory reports (compared to a 0-back task) following exposure to the trauma film. Following previous findings, we expect that brief wakeful rest resulted in enhanced consolidation to strengthen memory for the previous experiences compared to the 0-back task^26^. Brief wakeful rest has previously been shown to enhance subsequent memory performance and be associated with greater hippocampal processing compared to a simple cognitive task/presentation of new stimuli^10,26^. Further, these processes do not seem to rely on conscious rehearsal^27^ and wakeful rest may hence support consolidation processes similar to hippocampal replay identified during sleep and wakeful rest in rodents^31,32,37,38^.

Under a dual representation account of intrusive imagery^2,22^, enhanced consolidation would allow weak contextual representations formed at encoding to be strengthened and more strongly associated with the negative content of the traumatic experience, which would reduce the occurrence of intrusive retrieval. Our findings are also in line with previous work showing that enhanced processing of an experimental trauma following encoding (by carrying out a memory test) can reduce intrusions and facilitate deliberate memory performance^39^. The dissociable effects of wakeful rest versus a 0-back task on deliberate memory and intrusive memories are not consistent with a unitary view of trauma memory in which intrusive imagery and deliberate memory are strengthened or weakened in similar ways^5^.

Our results cannot be explained in terms of a simple distraction account^40^, which would predict fewer intrusive memory reports following the use of a vigilance task to distract attention and prevent rehearsal or consolidation. In addition, the comparable performance across conditions on deliberate recognition (tested a week later) also argues against a general disruption of memory through distraction^17^.

Our results are also not likely to be due to differences in emotion regulation during wakeful rest and completing the 0-back task. As described, to monitor thoughts during wakeful rest and the 0-back task, we administered the DSSQ following encoding and global DSSQ scores did not reveal any differences between conditions in any of the two experiments. For the trauma-related thoughts component of the DSSQ specifically, we observed a significant difference between conditions in Experiment 2 only, with more trauma-related thoughts in the wakeful rest condition compared to the 0-back condition. The latter finding could suggest that thinking about the videos following encoding was beneficial for reducing intrusions^39^. However, we did not find support for this interpretation in Experiment 1, and previous studies have highlighted that intentional recall is not necessary for the effects of wakeful rest on neutral memories^27^.

An alternative interpretation of our results could be that carrying out the 0-back task following encoding *increased* subsequent memory intrusions. Although we cannot rule out this possibility, the 0-back task is a simple task that requires sustained attention but does not place any heavy cognitive demands on visuospatial or verbal processing of the types that have previously been shown to alter the prevalence of subsequent memory intrusions. It has also been shown not to affect hippocampal or amygdalar activity following viewing of stressful videos^35^. Hence, we believe that the 0-back task is unlikely to interfere with post-encoding processing of the video content or exert significant influence on subsequent memory processes. In contrast, brief wakeful rest has been associated with enhanced subsequent memory compared to simple cognitive tasks with and without intentional rehearsal^26,27^.

How do our findings relate to studies showing that a visuospatial task after viewing traumatic footage can decrease intrusions compared to a verbal or no-task control condition?^14,15^ We suggest that different types of tasks following viewing might impair or facilitate consolidation of different memory representations. As presented in the Introduction, the dual representation account predicts that memory intrusions can be reduced either by weakening sensory or emotional representations of the trauma, or by strengthening contextual representations of it.

Hence, playing a visuospatial computer game might interfere with consolidation of perceptual/visuospatial aspects of the trauma film such as the sight of an injured person^15,16,25^. In contrast, neither the wakeful rest nor the vigilance task in the current study involved extensive visuo-spatial processing, and so are unlikely to have interfered with consolidation of perceptual representations. Thus we are left with the conclusion that consolidation of other aspects of the videos (e.g. context, gist, narrative) is differentially affected by wakeful rest or the vigilance task. This notion is consistent with studies showing that encoding of new information immediately following an event (compared to an empty inter-trial interval) reduces post-encoding hippocampal processing that aids memory^11^.

In contrast to the observed relative reduction in intrusive memory, we did not find significant differences between brief wakeful rest and 0-back tasks on deliberate memory performance for the traumatic material when tested after 1 week. This result contrasts with studies showing a benefit of wakeful rest on subsequent deliberate free recall of neutral information compared to a simple cognitive task^26^. It is possible that the lack of an effect on overall memory performance (d’) is due to the combination of using a recognition memory test with negative items, rather than the free recall test with neutral items used in a previous study^26^. Although hit rate in itself does not reflect memory performance, the increased hit rate after wakeful rest seen in our experiment 1 might correspond to increased performance in a free recall task. Experiment 2 (including negative and neutral clips) indicated that the increased false alarm rates are specific to negative items (Fig. 2d), similar to studies showing increased false alarm rates and a more liberal response bias for emotional stimuli^41–43^. Increases in deliberate memory performance after wakeful rest have also been shown for emotional materials in some previous studies^28,29,44^. Again, the absence of an effect in our study is hard to interpret, but may reflect differences in the emotionality of the stimuli used, which has an inverted U-shaped relationship to deliberate memory performance^45^. Thus brief wakeful rest may increase memory consolidation compared to a vigilance task, but whether or not this plays out in an increase in deliberate memory performance may depend on the nature of the memoranda and the type of test used.

From the dual representation theory, we would expect to see that wakeful rest would modulate memory intrusions and deliberate memory in opposite directions, but we did not see a change in deliberate memory. However, the observed condition × memory type interactions in both experiments does suggest that diary intrusions and deliberate memory was differentially affected by the post-encoding task (wakeful rest compared to 0-back).

Our results have clinical implications in highlighting the immediate aftermath of a trauma as an important period in which post-encoding processes can alter intrusive memory development. Providing a period in which trauma victims could clearly consolidate the event and its surrounding context might be beneficial in reducing related symptoms. This would be an alternative potential route to reducing intrusions than attempting to specifically disrupt consolidation of traumatic visual information^14,15^. However, we note that the level of arousal experienced in our experiments would be much less than that experienced in real life trauma. Thus, even with a rest period in which to encourage consolidation, the severe arousal and stress being experienced by the individual might disrupt consolidation processes nonetheless. Strategies that attempt to reduce arousal without affecting memory consolidation, as seen in therapeutic approaches such as eye movement desensitisation reprocessing^46^ (EMDR), might provide an optimal way to reduce traumatic memory disturbances. Nevertheless, our results suggest that strengthening hippocampal consolidation of experienced trauma might provide a potential route towards reducing the subsequent development of intrusive imagery.

In conclusion, our results suggest that strategies aimed at facilitating memory consolidation (in our case a period of wakeful rest compared to a similar period of a vigilance task) can reduce intrusive imagery and that these strategies have dissociable effects on intrusions and deliberate memory. The findings provide support for dual processing accounts of intrusive imagery, demonstrating that the expression of deliberate and intrusive memory can be manipulated independently. Furthermore, our results can contribute to furthering the mechanistic understanding of why psychotherapeutic interventions that elaborate on deliberate memory for the trauma can successfully reduce memory intrusions.

## Methods

### Experiment 1

#### Procedure

**Figure 3:**
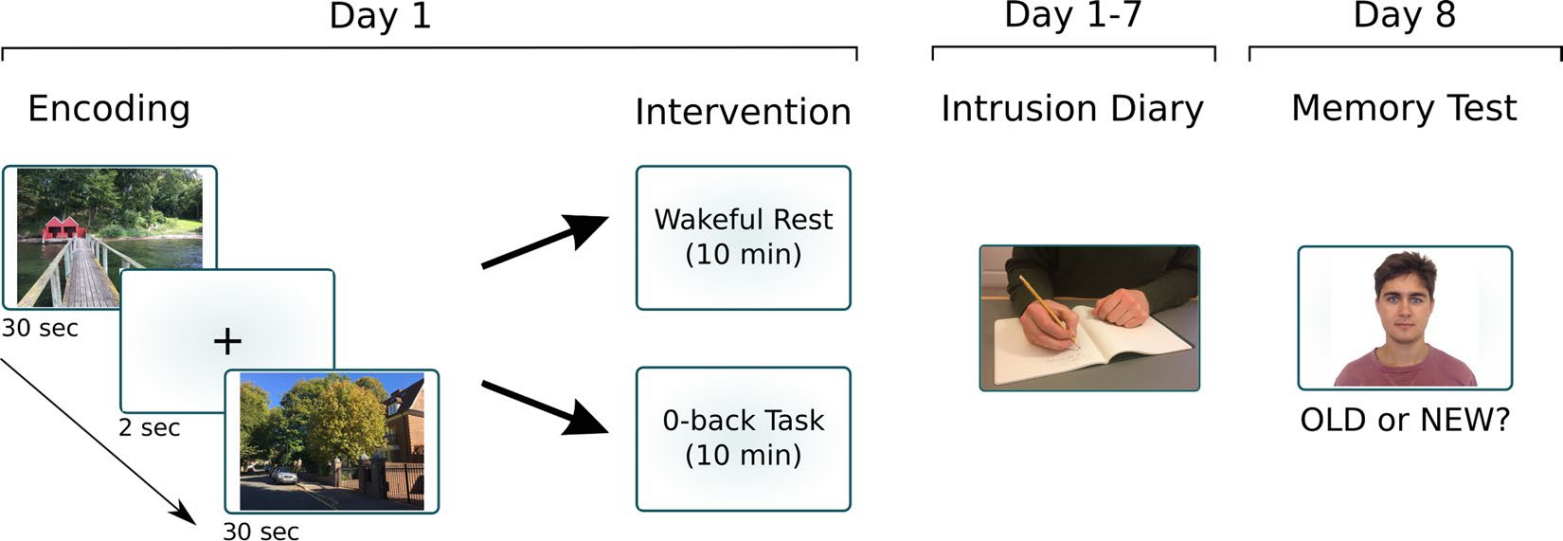
Experimental Procedure in Experiment 1. On day 1, participants viewed a ‘trauma film’ comprising 20 clips, each with a duration of 30 sec. Immediately following encoding, participants either received a period of wakeful rest (N = 21) or a vigilance (0-back) task (N = 19). In the week following encoding, participants kept a diary of clip-related spontaneous memories. On day 8, participants return for a recognition memory task.

On day 1, participants were randomly assigned to one of the two experimental conditions (brief wakeful rest or vigilance task) and completed questionnaires to measure trait and state anxiety (STAI) and current positive and negative mood states (PANAS). Participants next watched the trauma film and were instructed to imagine that they were at the scene, watching the events unfold in front of them (Fig. 3). Immediately after watching the clips, participants were required to either sit quietly for 10 minutes (brief wakeful rest group) or carried out a 10-minute 0-back task (vigilance task group). Next, participants again completed questionnaires to measure state anxiety (STAI), current positive and negative mood states (PANAS) and engagement, worry and trauma-related thoughts following encoding (Dundee Stress State Questionnaire). At the end of the session, participants were provided with instructions (both verbal and written) on keeping the intrusion diary.

Over the next 7 days, participants recorded any spontaneous memories about the trauma film using the intrusion diary. On their return 1 week later, information recorded in the intrusion diary was checked by the experimenter. Next, a recognition memory test was completed, during which cropped static images from the trauma film and new foil images were presented one at a time. On each test trial (Fig. 3), an image was presented in the centre of the screen and participants were required to respond OLD or NEW via button press as to whether they recognised previously seeing the image during encoding or if they thought the image was new, respectively.

Sample size: Based on an effect size of d = 0.91 from a previous study investigating post-encoding interventions and intrusions^15^, a sample size estimation was calculated using the G*power 3.1 software. This analysis indicated a required sample size of N = 20 in each group to achieve an 80% power level at α = 0.05.

Participants: Forty healthy volunteers (29 females, mean age = 22.8 years, SD = 3.36) were recruited from the university student population. The study was approved by the University College London Research Ethics Committee and participants provided written informed consent prior to taking part. The study was performed in accordance with relevant guidelines and regulations. Volunteers were informed about the nature of the study and were aware that they would view traumatic clips. Participation in the study was paid and participants with a history of psychiatric or neurological disorders were excluded from taking part.

### Materials

#### Trauma film

The trauma film consisted of 20 audio-visual clips involving traumatic and realistic events, collected from video sharing websites and containing graphic imagery involving actual or threatened death and serious injury. Each clip contained a clear narrative and had a duration of ~30 seconds. Clips were selected from a larger set of clips used in pilot experiments with the final subset selected on their ability to reliably induce intrusive memories. Similar clips have been used successfully to induce memory intrusions in a number of studies^12,13^.

#### Vigilance task

The vigilance task consisted of a verbal n-back task^47^ in which participants were shown single numbers (1–9) on a screen in a pseudo-randomised order. In the typical n-back, participants are required to attend to each number and make a response when the number on the screen matches the number in n positions backwards. We utilised a 0-back version of the task in which the numbers are presented in black font and participants respond via key press when the number on the screen appears in a different colour. Each number was presented for 1500 ms followed by an inter-trial interval consisting of fixation for 1000 ms. Participants were instructed to respond to the target stimulus as quickly as possible with the total time of the task being 10 minutes. The memory load for a 0-back task is light, requiring change-detection and memory for the rules.

#### Wakeful rest

Participants were instructed to sit quietly and relax until they were told that the session was over. They were not required to close their eyes and were not given any instructions as to what they could or could not think about but were simply asked to relax and let their mind wander as they wished. The room did not have any windows and was generally quiet and undisturbed.

#### Self-report questionnaires

Trait and state anxiety were measured using the State-Trait Anxiety Inventory^48^ (STAI). Each of the scales comprised 20 items with participants required to rate items on a 4-point scale, yielding a total score in the range of 20–80. Higher scores indicate greater anxiety. Positive and negative affect was measured using the Positive Affect and Negative Affect Scales^49^ (PANAS), yielding separate scores for positive and negative affect. The PANAS consists of 20 items where each is rated on a 5-point Likert scale.

Trauma-related thoughts for the clips were measured with the Dundee Stress State Questionnaire (DSSQ), which assesses subjective stress state symptoms related to mood, motivation and cognition^50^. Two measures were obtained from the DSSQ: a total score comprising the sum of scores on all items and a trauma-related thoughts subscore calculated from the sum of items assessing thoughts related to the presented videos (e.g. ‘I thought about the videos and how they made me feel’). This questionnaire was given to assess trauma-related thoughts participants might have experienced during rest or the vigilance task. Hence, this measure reflects the degree to which participants thought about the experimental trauma videos during the subsequent intervention with either wakeful rest or vigilance task.

#### Intrusion diary

Memory intrusions were recorded for one week using a pen and paper diary. Participants recorded any spontaneous memories they experienced over the week relating to the clips they had watched. Spontaneous memories were defined as memories that either occurred with no apparent reason or memories that were triggered by environmental stimuli. Participants were instructed to report all clip-related spontaneous memories in the diary, with a different entry for each memory specifying 1) a brief description of the memory intrusion, 2) in what situation the memory occurred and 3) which clip the memory was related to. If participants experienced no spontaneous memories during a day, they were instructed to still make an entry in the diary with a notification that they had zero spontaneous memories for that day.

#### Memory test

To test memory for the trauma film, 3–5 images were taken from each clip giving a total of 78 images. Each image was a cropped section of the full scene viewed during a clip (e.g., a person or object from a scene) to increase difficulty. In addition, 37 new images were taken from unseen clips showing similar events and used as foils at test.

#### Statistical analysis

Data from the trait anxiety questionnaire were analysed using an independent samples t-test (two-tailed) to examine group differences. State anxiety (STAI) and mood (PANAS) data were analysed using mixed ANOVAs with condition (wakeful rest, vigilance task) as a between participants factor and time (pre-, post-film) as a within participants factor. Deliberate memory performance was assessed using a signal detection method by dividing trials into hits (old images correctly recognised), misses (old images incorrectly judged as new), false alarms (new images incorrectly judged as old) and correct rejections (new images correctly judged as new). We then calculated d prime (d’) and response bias (c) scores using the formula d’ = z(H) − z(FA) and c = [z(H) + z(FA)]/2, where z(H) is the z-transform of the proportion of hits and z(FA) is the z-transform of the proportion of false alarms. Deliberate memory d’ scores were analysed using independent samples t-tests (two-tailed) to examine differences across conditions. All data were checked for assumptions of normality and for intrusion data where this assumption was violated, a log transformation was performed prior to analyses.

### Experiment 2

For Experiment 2, which used a within-participant design to increase statistical power, all materials and procedures were the same as Experiment 1 with the following exceptions:

#### Procedure

**Figure 4:**
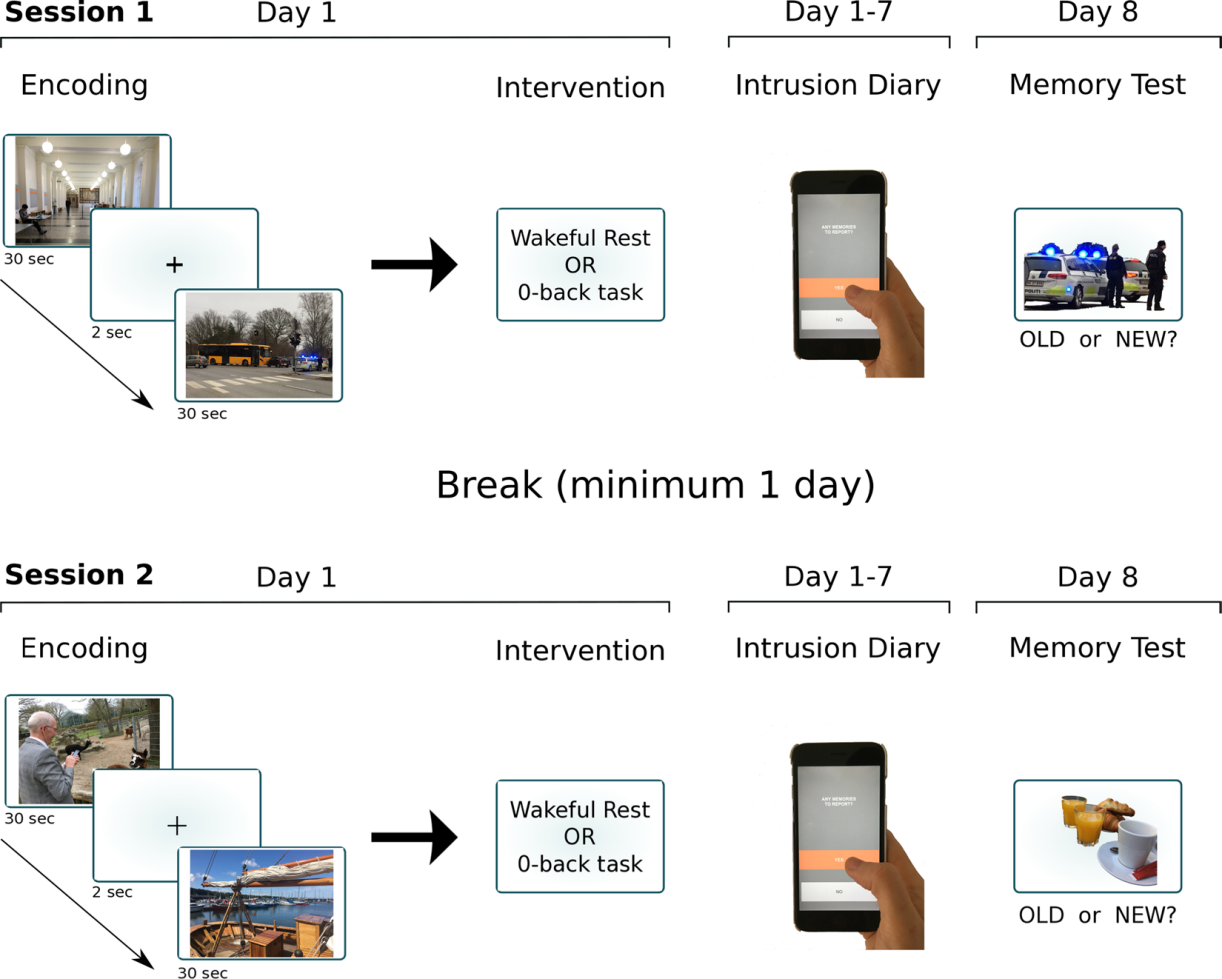
Experimental Procedure in Experiment 2. Experiment 2 was similar to Experiment 1 but used a within-participants design in which each participant completed two separate sessions with each session involving either wakeful rest or 0-back so that all participants completed both post-encoding procedures. Both negative and neutral clips were used. On day 8, participants returned for a recognition memory test and an intrusion provocation task. The order of videos and post-encoding procedure was counterbalanced across participants.

Participants were required to attend two full test sessions, with each session lasting 1 week and comprising initial trauma film encoding, a one-week intrusion diary and a follow up memory test (Fig. 4). Each encoding session involved watching 20 film clips, including 10 neutral and 10 negative clips. Clip selection for each encoding session was randomised from the total 40 clips to create two sets. In one session, participants were given brief wakeful rest following viewing the trauma film and, in the other test session, the vigilance task. The order in which the two sessions were performed was counterbalanced across participants (wakeful rest or vigilance task first) with a minimum of one day between completing the first session and starting the second session. The procedure of each test session was the same as Experiment 1 except that participants completed the additional intrusion provocation task at follow up prior to performing the recognition memory test.

Sample size: Effect sizes from Experiment 1 were used for sample size estimation for Experiment 2. Based on the effect sizes for the intrusion data, a sample size of N = 34 was estimated in order to achieve 80% power at α = 0.05. We decided to recruit a slightly larger sample than that suggested by the estimation based on our previous experiment, as Experiment 1 was a small study and we wanted to ensure that a potentially smaller effect in Experiment 2 could still be detected.

Participants: A total of 45 healthy volunteers (36 females, mean age = 22.70 years, SD = 4.38) were recruited from the university student population. Participants gave written informed consent prior to taking part and were debriefed and paid at the end of the study.

Statistical analysis: Self-report measures including the STAI-S and PANAS and deliberate memory data were analysed as in Experiment 1. The deliberate memory data were then analysed with a 2 × 2 repeated measures ANOVA with emotion (neutral, negative) and condition (wakeful rest, vigilance task) as within-subject factors. Intrusion data were analysed using a 2 × 2 repeated measures ANOVA with condition (wakeful rest, vigilance task) and test (diary, provocation) as within-subject factors. Further, a 2 × 2 repeated measures ANOVA for negative clips only was conducted with memory test (intrusions, deliberate memory) and condition (wakeful rest, vigilance task) as within-subject factors. All data were checked for assumptions and as the assumption of normality was violated for the intrusion data, a log-transform was carried out on these data prior to analyses.

### Materials

#### Trauma film

Forty short film clips were used in total, including the same 20 negative clips from Experiment 1 and a further 20 neutral clips. Neutral clips were similar in duration to the negative clips (~30 sec) and comprised everyday events such as people meeting at a café or buying groceries at a supermarket.

#### Intrusion diary

Intrusive memories were recorded via a mobile phone app that participants downloaded during day 1. The app was designed in-house and incorporated the same questions as the pen and paper diary used in Experiment 1. Participants were instructed on how to use diary app on their phones and were instructed to complete an entry whenever an intrusion occurred over the following week. For participants unable to download the app (n = 6), a pen and paper diary was used.

#### Memory test

Similar to Experiment 1, deliberate memory for the trauma films was tested via a recognition memory test. There were 3–5 pictures for each video, yielding a total of approximately 80 test pictures (40 negative and 40 neutral) and 40 foils (20 negative, 20 neutral; numbers vary slightly depending on the allocation of videos on the two sessions). Each image was a cropped section including a specific person or object from the entire scene, to increase difficulty. Foils were collected from other similar videos that were not included in the study.

#### Intrusion provocation task

Twenty blurred static images were created using a Gaussian Blur function (GIMP Software; Free Software Foundation, 2010) set between 40–70 pixels depending on the image, so that the features of the image were vaguely distinguishable. Images included one image from each of the trauma film clips. Images were presented for 2-sec each in a randomised order. Immediately after viewing the images, participants were instructed to sit quietly and relax for 2 minutes and, if they experienced any intrusive memories, were instructed to write down the memory on a sheet of paper.

## Data Availability

The datasets generated during and/or analysed during the current study are available from the corresponding author on reasonable request.
